# Functional Connectivity Changes in Behavioral, Semantic, and Nonfluent Variants of Frontotemporal Dementia

**DOI:** 10.1155/2018/9684129

**Published:** 2018-04-01

**Authors:** P. Reyes, M. P. Ortega-Merchan, A. Rueda, F. Uriza, Hernando Santamaria-García, N. Rojas-Serrano, J. Rodriguez-Santos, M. C. Velasco-Leon, J. D. Rodriguez-Parra, D. E. Mora-Diaz, D. Matallana

**Affiliations:** ^1^Departamento de Radiología e Imágenes Diagnósticas, Hospital Universitario San Ignacio, Bogotá, Colombia; ^2^Facultad de Medicina, Pontificia Universidad Javeriana, Bogotá, Colombia; ^3^Facultad de Ingeniería, Pontificia Universidad Javeriana, Bogotá, Colombia; ^4^Centro de Memoria y Cognición Intellectus, Hospital Universitario San Ignacio, Bogotá, Colombia; ^5^Departamento de Psiquiatría, Universidad Nacional de Colombia, Bogotá, Colombia

## Abstract

Frontotemporal dementia (FTD) affects behavior, language, and personality. This study aims to explore functional connectivity changes in three FTD variants: behavioral (bvFTD), semantic (svPPA), and nonfluent variant (nfvPPA). Seventy-six patients diagnosed with FTD by international criteria and thirty-two controls were investigated. Functional connectivity from resting functional magnetic resonance imaging (fMRI) was estimated for the whole brain. Two types of analysis were done: network basic statistic and topological measures by graph theory. Several hubs in the limbic system and basal ganglia were compromised in the behavioral variant apart from frontal networks. Nonfluent variants showed a major disconnection with respect to the behavioral variant in operculum and parietal inferior. The global efficiency had lower coefficients in nonfluent variants than behavioral variants and controls. Our results support an extensive disconnection among frontal, limbic, basal ganglia, and parietal hubs.

## 1. Introduction

A major objective in current clinical neuroscience research is to find new and more accurate neural footprints to improve the diagnosis and follow the progression of neurodegenerative disorders [1]. Frontotemporal dementia (FTD) is a group of clinically and pathologically heterogeneous diseases [2–4]. It has variants with different kinds of manifestations in behavior, language, metacognition, and personality. This clinical heterogeneity makes it difficult to obtain an accurate diagnosis [5].

FTD has been associated with regional atrophy in the frontal and temporal lobes [6]. It usually appears in the age group 45–64 years [7] with prevalence of 0.01–4.6 per 1000 persons [8]. Moreover, the clinical and genetic features are heterogeneous and there is still no treatment available for these conditions [4]. FTD encompasses three main phenotypes characterized by specific clinical symptoms. The behavioral variant FTD (bvFTD) is characterized by changes in personality [2], alteration in social cognition [9], disinhibition, and apathy. Nonfluent/agrammatic variant primary progressive aphasia (nfvPPA) is characterized by agrammatism and fluency impairment mainly [10]. Patients with the semantic variant (svPPA) have a loss of semantic knowledge and relative preservation of grammatical aspects of language and episodic memory [5]. A clinically similar linguistic variant, differentiated by the etiology, is the logopenic variant of PPA (lvPPA); it is an atypical variant of Alzheimer's disease with anomia, hesitant speech, and alterations in episodic memory [11].

Several biomarkers have been suggested to aid the clinical diagnosis and treatment. Neuroimaging biomarkers have been derived from structural magnetic resonance imaging (MRI), FDG-PET, SPECT, and functional MRI such as the resting state and functional activation imaging [12]. Structural MRI studies have consistently reported frontotemporal atrophy with a relative sparing of posterior cortical areas in bvFTD [13]. Semantic dementia involves a large area of the temporal lobe; nevertheless, there is a marked degeneration in the rostral fusiform gyrus and ventral temporal lobe bilaterally [14, 15]. In nfvPPA, imaging studies showed atrophy mainly involving the left inferior frontal lobe, insula, and premotor cortex [13, 16–18].

Another biomarker of FTD based on neuroimaging is resting-state fMRI [13, 19]. Resting-state fMRI can be used to show functionally connected brain networks by measuring synchronized time-dependent changes in blood oxygenation levels [20]. Prior research reported a reduction in limbic connectivity and the insula, putamen, anterior thalamus, and middle cingulate cortex in svPPA and bvFTD with respect to controls [21]. Another result showed an increased and diffused prefrontal hyperconnectivity, and it was significantly associated with apathy [21]. Longitudinal studies report a functional connectivity decrease over time in bvFTD between the supramarginal gyrus and the right frontoparietal network [22].

Recent studies showed that svPPA has a disrupted functional connectivity between the anterior temporal lobe [23, 24] and a broad range of regions including primary cortices (sulcus, Heschl's gyrus, precentral and postcentral gyri, and dorsal posterior insula (primary interoceptive cortex)) and auditory and visual association regions [25]. Both svPPA and bvFTD patients show a reduced functional connectivity in limbic areas of the executive network. However, svPPA patients also exhibit a reduced functional connectivity in the bilateral lateral prefrontal cortex and anterior cingulate [21]. In nfvPPA, previous studies have demonstrated compelling evidence that motor speech and grammatical deficits are associated with deficits in the left frontoinsular-striatal structures involved in speech production, a finding related to a reduced activation of a ventral portion of the left inferior cortex during attempts to understand grammatically challenging aspects of a sentence [26–29]. One study with resting-state fMRI analysis in nfvPPA showed connectivity changes in three subnetworks, namely, (a) the left inferior frontal gyrus and the left supplementary motor area, (b) inferior and superior parietal gyri between both hemispheres, and (c) striatum with the supplementary motor area in both hemispheres [30].

The functional connectivity among frontotemporal subvariants has been explored in a few studies. In the literature, usually, there are comparisons between controls and patients with bvFTD or with Alzheimer's disease [21, 31]. This study attempts to describe the alterations in functional connectivity networks among frontotemporal dementia variants to find specific connectivity alteration in each variant. First, we compared the functional connectivity of the whole brain among the variants. Second, topologic measures such as global efficiency, degree, path length, and clustering from each patient and between variants were compared.

## 2. Methods

### 2.1. Participants

Seventy-six patients with FTD were selected from Hospital Universitario San Ignacio including thirty-two healthy controls. The FTD diagnosis was initially made by a group of experts, and each case was individually reviewed at a multidisciplinary clinical meeting (neurologist, neuropsychologist, psychiatrist, and geriatrician). The sample included 50 patients with bvFTD, 14 with svPPA, and 22 with nfvPPA diagnosis. Patients were diagnosed with bvFTD based on recent guidelines [3]. These patients showed prominent changes in personality and social behavior as verified by a caregiver during their initial assessment. svPPA diagnosis were done based on international guidelines [18], and these patients included here had important semantic failures. Patients with nfvPPA have an evaluation by an expert in linguistic, and diagnosis was done based on international guidelines [11].

Control subjects were matched with bvFTD, svPPA, and nfvPPA patients (see Table 1). Matching criteria were gender, age, and years of education. An analysis of variance with Holm-Sidak's multiple comparison test did not show differences among groups to age and years of education. Subjects were recruited from a larger pool of volunteers who did not have a neurodegenerative disease diagnosis or psychiatric disorders. All the participants provided written informed consent in accordance with the institutional review board of the Hospital Universitario San Ignacio and Pontificia Universidad Javeriana.

### 2.2. Cognitive and Behavioral Assessment

Neuropsychological evaluation was performed in patients and controls. The test battery included screening tests, Montreal Cognitive Assessment (MoCA) [32, 33], mini-mental state examination (MMSE), and INECO Frontal Screening (IFS) test [34]. Verbal inhibitory control was measured by Hayling test [35]. We used Wisconsin Card Sorting Test (WCST) modified to evaluate executive functions [36]. Rey-Osterrieth complex figure (ROCF) test was employed to assess visuomotor skills [37]. Frontal system behavior scale (FrSBe) [38] was used to measure behavioral changes. This test had two sections investigating premorbid or current behavior. Apathy, inhibition, and dysexecutive function subscales were estimated by FrSBe.

Verbal and design fluency tests were used to assess recall, self-monitoring and cognitive flexibility strategies, phonological (words with P and M), and semantic fluency (animals and fruits) [39]. Finally, proverbs test [40] was used to assess verbal comprehension.

### 2.3. Image Acquisition

Images from patients with FTD and controls were obtained using a Philips Achieva 3T scanner with a 16-channel SENSE coil. The anatomical and 3D T1-weighted images had the following parameters: TR = 7.9 ms, TE = 3.8 ms, acquisition matrix = 220 × 220, voxel size = 0.5 × 0.5 × 0.5 mm, and 310 slices, and these images were resliced to 1 × 1 × 1 mm. The blood oxygenation-dependent sequences of the entire brain were acquired in 25 axial slices by using an echoplanar imaging sequence TR = 2000, TE = 30 ms, and voxel size = 2.3. The fMRI lasted 6 minutes and the instruction to the patient was to keep their open eyes.

### 2.4. Data Analysis

#### 2.4.1. Behavioral Analysis

Demographic information and scores from clinical tests were compared among groups with ANOVA tests and post hoc test for multiple comparisons and correction of *p* values by Sidak.

#### 2.4.2. Processing and Analysis

Preprocessing was performed with a combination of the Statistical Parametric Mapping [39] software (http://www.fil.ion.ucl.ac.uk/spm/software/spm12/) (Wellcome Department of Cognitive Neurology, University College London, UK), the Resting-State fMRI Data Analysis Toolkit (REST) version 1.8 (http://www.restfmri.net) [40], and Data Processing Assistant for Resting-State fMRI (DPABI) version 2.1 (http://rfmri.org/DPABI).

#### 2.4.3. Resting-State Preprocessing

The main preprocessing procedure was done with DPABI [41], and the pipeline was (1) removal of the first 10 time points, (2) slice timing, (3) head motion correction, (4) nonlinear registration of the high-resolution T1 structural images to the Montreal Neurological Institute (MNI) template, in which T1 structural images were segmented as white matter, gray matter, and cerebrospinal fluid using a new segment algorithm with DARTEL (diffeomorphic anatomical registration through exponentiated lie algebra), (5) smoothing with a 6 mm full-width-half-maximum Gaussian kernel, (6) removal of the linear trend of the time series, (7) temporal band-pass filtering (0.01–0.08 Hz) to decrease the effects of low-frequency drifts and high-frequency noise, and (8) linear detrending and nuisance signal removal, white matter, cerebrospinal fluid, global signal, 6-head motion parameters, 6-head motion parameters at one time point earlier, and the 12 corresponding squared items (Friston 24-parameter model as covariates) via multiple regression. The general pipeline was reported in another research [19].

#### 2.4.4. Seed-Based Analysis

The functional connectivity was estimated with a seed-based analysis. Regions of interest (ROIs) or seeds were selected according to automated anatomical labeling (AAL) atlas [42]. The diameter of the sphere ROI was 10 mm (approximately 27 cubic voxels). The seed analysis only included the brain. Pearson correlation coefficients were calculated between the mean time course of the ROI and the time courses for all other brain voxels. Fisher's *z* transform analysis was applied to the Pearson correlation coefficients to obtain an approximate normal distribution to enable the subsequent statistical analysis.

#### 2.4.5. Network-Based Analysis

Global differences in interconnected network components between patients and controls were examined with an *F*-test by network-based statistics (NBS) [43] based on 10,000 permutations. The *p* value threshold was set at 0.01 and it was corrected by family-wise error (FWE). Contrasts between groups were bvFTD versus controls, nfvPPA + svPPA versus controls, and bvFTD versus nfvPPA + svPPA.

#### 2.4.6. Graph Theory Analysis

In a secondary analysis, the connectivity metrics such as path length, degree, cluster, and global efficiency were estimated by the Brain Connectivity Toolbox [44]. The correlation between ROIs was graphically represented by a collection of nodes and edges (nodes represent anatomical elements like brain regions and the edges represent the connectivity between those regions). In these graphs, the degree represents the number of edges connected to a node. A cluster is an extension of local interconnectivity. The path length is the number of edges that connect a node with another node, and global efficiency measures the ability of a network to transmit information at a global level. Network centrality (NC) measures the numbers of the shortest paths that go through a node and link the other node pairs across the network [45]. It indicates the importance of a node for efficient communication and integration across a network [45]. Several studies have already used NC (also called “betweenness centrality”) to identify changed connections in disconnection syndromes [31, 46, 47]. Finally, an analysis of variance between groups with connectivity metrics was used to evaluate differences among groups.

## 3. Results

An analysis of variance (ANOVA) on MOCA, MMSE, ROFC, semantic and phonological and fluency, and proverb scores yielded significant variation among groups (*p* < 0.05 in all cases) (see Table 2). There were no differences among variants (bvFTD, svPPA, and nfvPPA) on FrSBe before or currently (*p* > 0.05 in all cases). A post hoc test with Sidak correction showed higher scores in bvFTD than nfvPPA and svPPA on MOCA, semantic, and phonological fluency (*p* < 0.05 in all cases). Besides, the scores on MMSE and IFS were significantly higher in bvFTD with respect to nfvPPA (*p* < 0.05 in all cases). There were no differences among variants on Hayling, FrSBe, errors in WSCT, ROFC, and proverbs.

**Figure 1:**
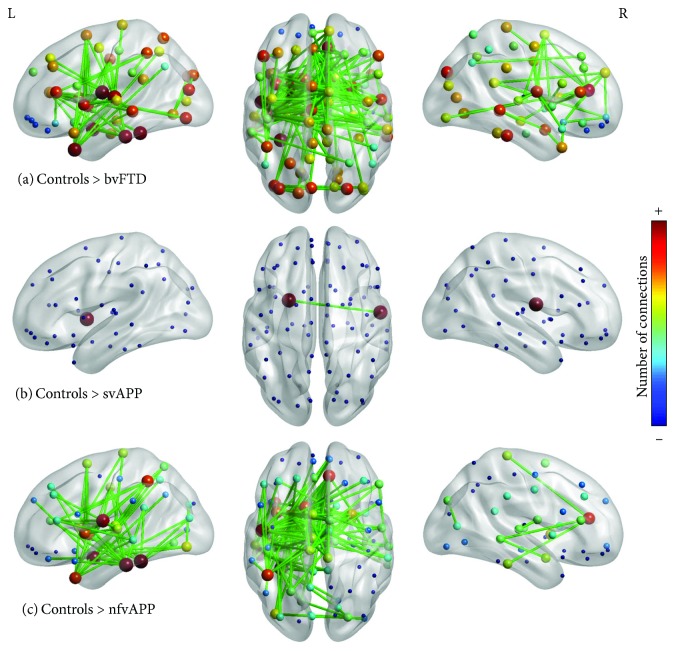
NBS results between controls and FTD variants. The edges are the result of *F*-test between groups. To nodes, the color corresponds to disconnection number.

The results with network-based statistics showed significant differences between the control group and bvFTD, svPPA, nfvPPA, and svPPA + nfvPPA groups. The first comparison between control and bvFTD (Figure 1(a)) showed significant differences in networks with nodes mainly in the left hemisphere in the frontal and temporal lobes (Table S1). Almost 15 nodes located in the left hemisphere in different regions (anterior and posterior) had a higher disconnection than controls. Moreover, in the right hemisphere, the nodes disconnected were anterior cingulate cortex, inferior temporal gyrus, superior occipital gyrus, middle temporal gyrus, putamen, amygdala, inferior frontal triangular gyrus, and fusiform gyrus.

With respect to results with linguistic variants, there were more differences in nfvPPA than svPPA. The comparison between control and svPPA groups showed only connectivity differences between the right operculum and the left putamen (Figure 1(b) and Table S2). The analysis between control and nfvPPA showed differences mainly in the left hemisphere (Figure 1(c) and Table S3). The nodes with disconnection were the inferior temporal gyrus, fusiform gyrus, amygdala, operculum, temporo-parieto-occipital junction, caudate nuclei, inferior parietal gyrus, putamen, and insula. Also, in the right hemisphere, there were nodes disconnected such as the anterior cingulate and the putamen.

**Figure 2:**
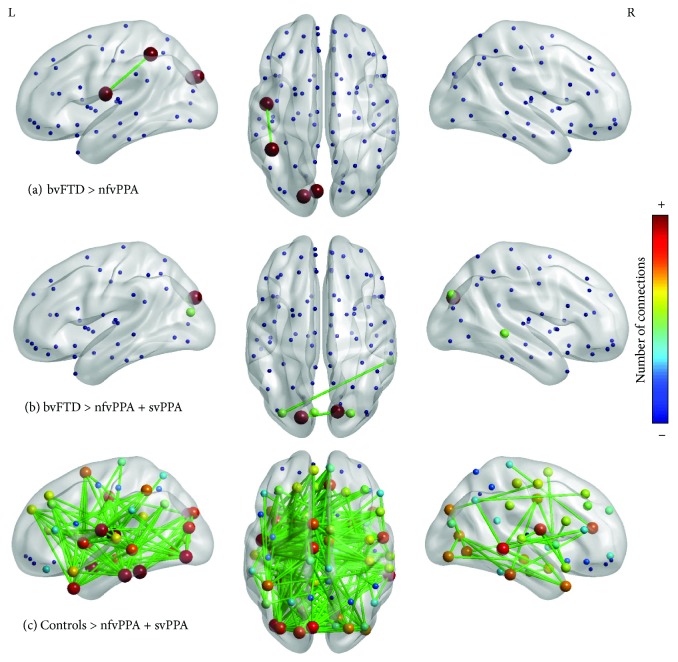
NBS results between FTD variants and controls.

The analysis between FTD variants showed differences between bvFTD and nfvPPA into the left hemisphere to the connection between operculum with parietal and cuneus left with occipital superior gyrus (Figure 2(a)). There were no differences between controls and svPPA patients. The comparison between bvFTD and all patients with linguistic alterations showed a disconnection of the left superior occipital, left middle occipital, and right middle temporal gyri (Figure 2(b)). Finally, the comparison between controls and all linguistic variants (Figure 2(c)) showed a major disconnection in Heschl's left gyrus, left amygdala, left fusiform, left inferior temporal gyrus, right middle temporal gyrus, and left temporal pole (Table S4).

**Figure 3:**
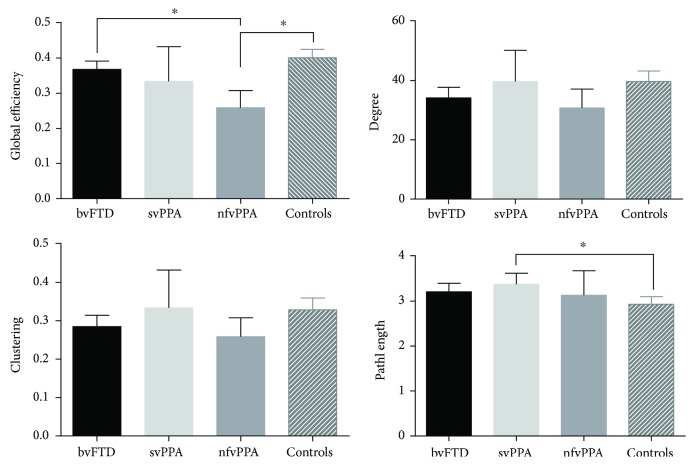
Mean bar of metrics from graph theory analysis by each group (global efficiency, degree, clustering, and path length). The bar represents the mean and error bars are a 95% confidence interval. ^∗^Significantly different with *p* < 0.05.

**Figure 4:**
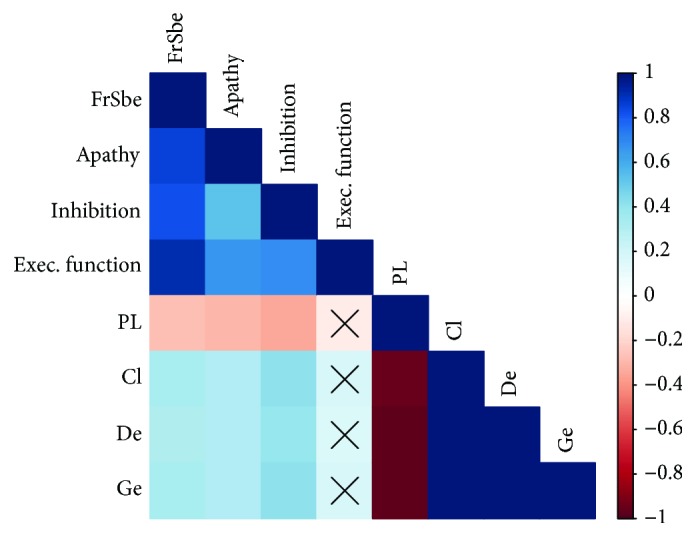
Matrix correlation among topological measures and clinical scores. The bar color indicates the Pearson value coefficient. Symbol X indicates *p* values > 0.05 with BH correction; FrSBE: total FrSBE currently; apathy: FrSBE apathy currently; inhibition: FrSBE inhibition currently; Exec. function: FrSBE dysexecutive functions currently; PL: path length; Cl: clustering; De: degree; Ge: global efficiency.

An analysis of variance based on topological metrics showed differences in global efficiency (*F*(3, 65) = 11.48, *p* < 0.001) and path length (*F*(3, 65) = 3.27, *p* = 0.026) (Figure 3). In the post hoc test, the global efficiency in bvFTD was significantly higher than nfvPPA; in addition, this measure was higher in controls than nfvPPA patients (*p* < 0.05 in both cases). Finally, we computed Pearson correlations, with correction for multiple correlational analysis [48], between topological metrics and clinical scores in all patients (Figure 4). We found significant associations of topological measures with FrSBe scores related to current behavior. The path length had significant and negative correlations with total FrSBe (*r* = −0.27), apathy (*r* = −0.3), and inhibition (*r* = −0.34). The clustering had significant and positive correlations with total FrSBe (*r* = 0.33), apathy (*r* = 0.3), and inhibition (*r* = 0.41). Also, the degree had similar correlations with total FrSBe (*r* = 0.32), apathy (*r* = 0.31), and inhibition (*r* = 0.4). Finally, the global efficiency had positive correlations with total FrSBe (*r* = 0.33), apathy (*r* = 0.31), and inhibition (*r* = 0.42).

### 3.1. Discussion

The study on connectivity based on resting-state functional MRI has the potential to identify differences among variants of FTD. The present study offers some contributions to understand the alterations in connectivity based on changes in networks and topological metrics. The approach based on network analysis showed more accuracy to detect differences than topological metrics of the whole brain with weighted matrices.

In this study, the bvFTD has a bilateral disconnection with a major tendency to nodes into the left hemisphere. Asymmetric results were reported in other studies, for example, a decrease in connectivity in the left frontoparietal network in bvFTD has been reported in comparison with controls [22]. Also, a decrease in connectivity between the right superior temporal gyrus and cuneal cortex was showed in bvFTD with respect to Alzheimer's disease [49]. Our results showed an extended bilateral disconnection between the frontal and limbic areas and the basal ganglia. A decrease between the frontal and limbic hubs was reported in another study [21]; this alteration could be associated with the disruption between affective and self-referential brain systems [21]. Also, the present results show alterations in the cingulum and insula network bilaterally. The cingulum has been associated with motivation and behavior control [50]. The anterior insula is a network hub to human emotional awareness and behavioral guidance networks [51]. Finally, in this report, the analysis supports alteration in posterior nodes in bvFTD, namely, there were disconnections in the middle occipital, inferior, and middle temporal gyri. Alterations in posterior regions in FTD are not frequent but have been reported previously [52].

The results support a connectivity decrease in linguistic variants in comparison with controls. The number of disconnected nodes was higher in nfvPPA than svPPA. In svPPA, the disconnection in the network between putamen and operculum has not been reported previously. However, one study reported atrophy in the putamen in svPPA [53], and the operculum has been associated with phonological processes that support reading [54]. In nfvPPA, a disconnection was found in networks involving hubs such as prerolandic areas and basal ganglia, regions related with speech production and syntactic process [55–57]. The topological metrics, global efficiency, and path length were useful to discriminate linguistic variants since global efficiency allows a differentiation between nfvPPA and bvFTD while path length differentiates svPPA and controls. Similar results were reported in a recent study; the path length in svPPA was higher in comparison with controls and similar to Alzheimer's disease patients, and it was correlated with the disease progression [58].

There was similarity among FTD variants in both clinical and neuroimaging analyses. Also, in this study, there were no differences between the linguistic variants (svPPA and nfvPPA). Nevertheless, nfvPPA was the variant with more differences than svPPA, both as the network analysis as topological metrics with respect to bvFTD. nfvPPA showed a worse measure in global efficiency and tends to have more degree and clustering than svPPA and bvFTD.

The behavioral changes measured by FrSBe did not show differences among variants. This result could indicate the presence of behavioral disturbances between linguistic variants and can support the presence of frontal alterations in nfvPPA and bvFTD. All patients had important behavioral changes in FrSBe scores related to premorbid and current behavior. However, only the current scores in apathy and inhibition (FrSBE subscales) were associated with topological measures. Therefore, global changes in functional connectivity could be associated with the presence of disturbances in behavior at least in these variants. The behavioral disturbances have been more reported in svPPA than nfvPPA [59–61]. Only one study reported behavioral changes in nfvPPA, and these behavioral changes were similar to Alzheimer's disease [62].

The limitations of this study are related to sample size, use of topological metrics from weighted matrices, and AAL atlas to create the seeds. The reduced sample size of nfvPPA was due to requirement of a second evaluation by an expert in order to exclude lvPPA. According to several reports, lvPPA is associated with the Alzheimer's variant [63–65]. With respect to topological metrics, these correspond to general measures from graph theory [44]. Both acquisition and image preprocessing could affect the analysis and measures. However, there is no gold standard method and applied protocols similar to those used in previous studies [66]. Finally, some studies show a scale effect in graph analysis related to the number of nodes [67–69], but we used AAL standard atlas to make our results comparable with those from other studies.

In conclusion, our result supports the use of global metrics from graph theory and network analysis to explore differences among some FTD variants. The nfvPPA showed more alterations in networks and global metrics than other variants, and also, alterations in bvFTD involve hubs in frontal lobes, limbic lobes, and basal ganglia. However, there are no differences between svPPA and nfvPPA in either NBS or topological measures. This preliminary study among variants in FTD allows us to identify several hubs and networks, and these can be used in the future to build biomarkers based on fMRI. Finally, the functional connectivity was associated with disturbances in behavior. New studies should explore the association among different biomarkers from multimodal neuroimaging, such as structural and functional connectivity, in order to obtain increased accuracy about networks with changes or alterations due to early onset dementia.

## Figures and Tables

**Table 1:** Sociodemographic characteristics.

Group	bvFTD	svPPA	nfvPPA	Controls	*p* value	Post hoc
Number (*n*)	50	14	12	32	—	—
Gender (F/M)	17/23	7/7	5/7	12/20	—	—
Age	65.85 (8.1)	60.3 (7.65)	63.63 (6.87)	61.25 (7.28)	0.02	ns
Disease duration (years)	7.27 (5.89)	5.85 (3.15)	4.28 (2.5)	—	—	—
Education (years)	12.92 (4.66)	12.3 (5.85)	11.62 (6.32)	14.4 (5.13)	0.33	ns

ns: no significant difference with Holm-Sidak.

**Table 2:** Clinical findings in patients and healthy controls.

	Controls	nfvPPA	svPPA	bvFTD	*p* value	Post hoc
MOCA	26.32 (2.57)	8.73 (7.26)	8.8 (6.58)	15.61 (7.53)	<0.001	1, 2, 3
MMSE	28.86 (1.27)	16.9 (6.92)	16.67 (7.66)	22.47 (6.5)	<0.001	1, 2, 3
IFS	22.3 (3.37)	6.20 (6.06)	10.8 (6.94)	10.7 (6.76)	<0.001	1, 2
Hayling	—	22.1 (11.09)	18 (13.52)	24.19 (12.66)	0.607	—
Errors WSCT	10.64 (8.14)	28 (7.77)	29.63 (8.88)	21.5 (10.18)	<0.001	1
ROFC	32.66 (5.05)	17.2 (11.86)	27.89 (9.35)	20.79 (12.26)	<0.001	1
FrSBe before	—	77.13 (18.63)	74.91 (29.39)	71.67 (18.79)	0.136	—
FrSBe currently	—	121.47 (32.22)	130.73 (42.66)	129.15 (31.65)	0.449	—
FrSBe apathy before	—	21.69 (4.92)	21 (10.28)	21.14 (7.57)	0.969	—
FrSBe apathy currently	—	46.46 (11.44)	46.55 (16.9)	44 (12.46)	0.76	—
FrSBe inhibition before	—	23.15 (5.68)	22.55 (9.17)	20.65 (5.36)	0.391	—
FrSBe inhibition currently	—	28.54 (7.66)	31.18 (10.05)	32.46 (11.46)	0.516	—
FrSBe DE before	—	32.08 (11.95)	31.27 (13.14)	29.38 (9.62)	0.702	—
FrSBe DE currently	—	50.92 (16.09)	52.55 (17.31)	53.95 (15.28)	0.833	—
Semantic fluency	16.68 (3.58)	5.91 (2.86)	4.20 (3.47)	10.45 (5.26)	<0.001	1, 2, 3
Phonological fluency	14.93 (5.05)	4.69 (3.31)	3.83 (2.79)	9.75 (5.47)	<0.001	1, 2, 3
Proverbs	8.54 (2.05)	2.53 (3.06)	1.4 (2.37)	4.09 (3.49)	<0.001	1

Mean and standard deviation were reported. *p* value from ANOVA. FrSBe DE: FrSBe dysfunction executive; nfvPPA: primary nonfluent aphasia; svPPA: semantic dementia; BV: behavioral variant; post hoc with Holm-Sidak (<0.05); 1: controls ≠ (bvFTD or svPPA or nfvPPA); 2: bvFTD ≠ nfvPPA; 3: bvFTD ≠ svPPA; 4: svPPA ≠ nfvPPA.

## Supplementary Materials

Supplementary 1 Table S1: networks with changes in bvFTD in comparison with controls.

Supplementary 2 Table S2: networks with changes in svPPA in comparison with controls.

Supplementary 3 Table S3: networks with changes in nfvPPA in comparison with controls.

Supplementary 4 Table S4: networks with changes in nfvPPA + svPPA in comparison with controls.
