# Supplementary material for: Functional Connectivity Changes in Behavioral, Semantic, and Nonfluent Variants of Frontotemporal Dementia
Source: Behav Neurol. 2018 Apr 1;2018:9684129. doi: 10.1155/2018/9684129 (PMC5902123; doi:10.1155/2018/9684129)
Supplement: Supplementary 2 — Table S2: networks with changes in svPPA in comparison with controls. [file 9684129.f2.docx]

**Supplementary Table S2.** Results with NBS in the contrast svPPA and controls. The rows indicate nodes pairs with a significantly difference between previous groups.

| Node 1 | Node 2 | T-test |
| --- | --- | --- |
| Rolandic_Oper_R | Putamen_L. | 3.44 |
| Amygdala_L | Cerebelum_8_L. | 3.67 |
| Cuneus_L | Cerebelum_8_L. | 5.53 |
| Cuneus_R | Cerebelum_8_L. | 4.58 |
| Occipital_Sup_L | Cerebelum_8_L. | 4.07 |
| Occipital_Sup_R | Cerebelum_8_L. | 4.18 |
| Occipital_Mid_L | Cerebelum_8_L. | 4 |
| Occipital_Mid_R | Cerebelum_8_L. | 3.63 |
| Parietal_Sup_R | Vermis_6. | 3.75 |
