# Supplementary material for: Functional Connectivity Changes in Behavioral, Semantic, and Nonfluent Variants of Frontotemporal Dementia
Source: Behav Neurol. 2018 Apr 1;2018:9684129. doi: 10.1155/2018/9684129 (PMC5902123; doi:10.1155/2018/9684129)
Supplement: Supplementary 3 — Table S3: networks with changes in nfvPPA in comparison with controls. [file 9684129.f3.docx]

**Supplementary Table S3.** Results with NBS in the contrast nfvPPA and controls. The rows indicate nodes pairs with a significantly difference between previous groups.

| Node 1 | Node 2 | T-test |
| --- | --- | --- |
| Rolandic_Oper_L | Parietal_Inf_L. | 5.88 |
| Insula_L | Parietal_Inf_L. | 4.4 |
| Amygdala_L | Parietal_Inf_L. | 4.31 |
| Parietal_Inf_L | SupraMarginal_L. | 5.22 |
| Rolandic_Oper_L | SupraMarginal_R. | 6.35 |
| Insula_L | SupraMarginal_R. | 5.14 |
| Cingulum_Ant_R | Precuneus_L. | 5.35 |
| Amygdala_L | Precuneus_L. | 4.22 |
| Cingulum_Ant_R | Paracentral_Lobule_R. | 4.76 |
| Amygdala_L | Paracentral_Lobule_R. | 4.19 |
| Precentral_R | Caudate_L. | 4.41 |
| Supp_Motor_Area_L | Caudate_L. | 4.42 |
| Supp_Motor_Area_R | Caudate_L. | 4.3 |
| Fusiform_L | Caudate_L. | 5.21 |
| Paracentral_Lobule_L | Caudate_L. | 5.32 |
| Paracentral_Lobule_R | Caudate_L. | 4.11 |
| Paracentral_Lobule_L | Caudate_R. | 4.21 |
| Rolandic_Oper_L | Putamen_L. | 5.42 |
| Supp_Motor_Area_L | Putamen_L. | 4.74 |
| Supp_Motor_Area_R | Putamen_L. | 4.18 |
| Fusiform_L | Putamen_L. | 5.77 |
| Paracentral_Lobule_R | Putamen_L. | 4.22 |
| Rolandic_Oper_L | Putamen_R. | 5.45 |
| Supp_Motor_Area_L | Putamen_R. | 4.51 |
| Fusiform_L | Putamen_R. | 4.21 |
| Paracentral_Lobule_L | Putamen_R. | 4.13 |
| Rolandic_Oper_L | Pallidum_L. | 4.16 |
| Cingulum_Ant_R | Pallidum_L. | 4.3 |
| Fusiform_L | Thalamus_L. | 4.79 |
| Fusiform_L | Thalamus_R. | 4.28 |
| Cingulum_Ant_L | Heschl_L. | 4.56 |
| Postcentral_L | Heschl_L. | 4.31 |
| Parietal_Inf_L | Heschl_L. | 4.32 |
| Paracentral_Lobule_L | Heschl_L. | 4.1 |
| Rolandic_Oper_L | Temporal_Sup_L. | 5.75 |
| Rolandic_Oper_R | Temporal_Sup_L. | 4.15 |
| Insula_L | Temporal_Sup_L. | 5.91 |
| Insula_R | Temporal_Sup_L. | 4.76 |
| Rolandic_Oper_L | Temporal_Sup_R. | 4.24 |
| Rolandic_Oper_R | Temporal_Sup_R. | 4.5 |
| Insula_L | Temporal_Sup_R. | 4.31 |
| Frontal_Inf_Tri_L | Temporal_Mid_L. | 4.18 |
| Amygdala_L | Temporal_Mid_L. | 4.88 |
| Frontal_Inf_Tri_R | Temporal_Mid_R. | 4.19 |
| Amygdala_L | Temporal_Mid_R. | 5.4 |
| Amygdala_R | Temporal_Mid_R. | 4.22 |
| Cingulum_Mid_L | Temporal_Pole_Mid_L. | 4.48 |
| Amygdala_L | Temporal_Pole_Mid_L. | 4.5 |
| Occipital_Mid_L | Temporal_Pole_Mid_L. | 4.26 |
| Angular_L | Temporal_Pole_Mid_L. | 4.68 |
| Caudate_L | Temporal_Pole_Mid_L. | 4.48 |
| Putamen_L | Temporal_Pole_Mid_L. | 6.21 |
| Putamen_R | Temporal_Pole_Mid_L. | 4.56 |
| Thalamus_L | Temporal_Pole_Mid_L. | 5.31 |
| Thalamus_R | Temporal_Pole_Mid_L. | 4.84 |
| Frontal_Sup_L | Temporal_Inf_L. | 4.25 |
| Frontal_Med_Orb_R | Temporal_Inf_L. | 4.38 |
| Cingulum_Ant_L | Temporal_Inf_L. | 4.59 |
| Cingulum_Ant_R | Temporal_Inf_L. | 5.11 |
| Cingulum_Mid_L | Temporal_Inf_L. | 5.09 |
| Cingulum_Post_L | Temporal_Inf_L. | 6.77 |
| Cingulum_Post_R | Temporal_Inf_L. | 4.65 |
| Hippocampus_L | Temporal_Inf_L. | 5.6 |
| Amygdala_L | Temporal_Inf_L. | 6.57 |
| Amygdala_R | Temporal_Inf_L. | 4.51 |
| Caudate_L | Temporal_Inf_L. | 5.94 |
| Caudate_R | Temporal_Inf_L. | 4.87 |
| Putamen_L | Temporal_Inf_L. | 5.93 |
| Putamen_R | Temporal_Inf_L. | 4.13 |
| Thalamus_L | Temporal_Inf_L. | 4.5 |
| Thalamus_R | Temporal_Inf_L. | 4.24 |
| Temporal_Pole_Sup_L | Temporal_Inf_L. | 4.18 |
| Cingulum_Ant_R | Temporal_Inf_R. | 5.05 |
| Amygdala_L | Temporal_Inf_R. | 4.75 |
| Caudate_L | Temporal_Inf_R. | 4.42 |
| Putamen_L | Temporal_Inf_R. | 4.17 |

**Table S4.** Networks with changes in nfvPPA+svPPA in comparison with controls

| Node 1 | Node 2 | T-test |
| --- | --- | --- |
| **Precentral_R** | Rolandic_Oper_L. | 3.7 |
| **Frontal_Mid_L** | Rolandic_Oper_L. | 3.56 |
| **Frontal_Mid_R** | Rolandic_Oper_L. | 4.23 |
| **Frontal_Inf_Oper_R** | Rolandic_Oper_L. | 3.16 |
| **Rolandic_Oper_L** | Rolandic_Oper_R. | 3.26 |
| **Frontal_Sup_L** | Olfactory_L. | 3.12 |
| **Olfactory_L** | Frontal_Sup_Medial_L. | 3.18 |
| **Olfactory_L** | Frontal_Sup_Medial_R. | 3.46 |
| **Olfactory_R** | Rectus_L. | 3.23 |
| **Frontal_Med_Orb_R** | Rectus_L. | 3.41 |
| **Precentral_R** | Insula_L. | 3.48 |
| **Frontal_Mid_R** | Insula_L. | 3.17 |
| **Rolandic_Oper_R** | Insula_L. | 4.26 |
| **Supp_Motor_Area_L** | Insula_L. | 3.48 |
| **Supp_Motor_Area_R** | Insula_L. | 3.73 |
| **Insula_L** | Insula_R. | 4.23 |
| **Rolandic_Oper_L** | Cingulum_Ant_L. | 3.98 |
| **Olfactory_L** | Cingulum_Ant_R. | 3.24 |
| **Rolandic_Oper_L** | Cingulum_Mid_L. | 4.14 |
| **Rolandic_Oper_R** | Cingulum_Mid_L. | 3.42 |
| **Rolandic_Oper_L** | Cingulum_Mid_R. | 4.72 |
| **Frontal_Sup_Medial_L** | Cingulum_Post_L. | 3.21 |
| **Frontal_Sup_Medial_R** | Cingulum_Post_L. | 3.14 |
| **Hippocampus_L** | ParaHippocampal_L. | 3.82 |
| **Hippocampus_R** | ParaHippocampal_L. | 4.45 |
| **Hippocampus_L** | ParaHippocampal_R. | 3.52 |
| **Hippocampus_R** | ParaHippocampal_R. | 3.52 |
| **Precentral_R** | Amygdala_L. | 3.54 |
| **Frontal_Sup_L** | Amygdala_L. | 4.11 |
| **Frontal_Sup_R** | Amygdala_L. | 3.79 |
| **Frontal_Mid_L** | Amygdala_L. | 3.54 |
| **Frontal_Mid_R** | Amygdala_L. | 4.26 |
| **Frontal_Inf_Orb_R** | Amygdala_L. | 3.46 |
| **Frontal_Sup_Medial_L** | Amygdala_L. | 3.67 |
| **Frontal_Sup_Medial_R** | Amygdala_L. | 3.91 |
| **Frontal_Mid_L** | Amygdala_R. | 3.19 |
| **ParaHippocampal_R** | Amygdala_R. | 3.11 |
| **Cingulum_Mid_L** | Cuneus_L. | 3.17 |
| **Calcarine_R** | Cuneus_L. | 3.42 |
| **Rolandic_Oper_L** | Lingual_L. | 3.29 |
| **Rolandic_Oper_R** | Lingual_L. | 3.7 |
| **Cingulum_Ant_R** | Lingual_L. | 3.15 |
| **Amygdala_L** | Lingual_L. | 3.17 |
| **Calcarine_L** | Lingual_L. | 3.68 |
| **Calcarine_R** | Lingual_L. | 3.72 |
| **Cuneus_L** | Lingual_L. | 3.37 |
| **Cingulum_Ant_R** | Lingual_R. | 3.35 |
| **Calcarine_L** | Lingual_R. | 3.25 |
| **Cuneus_L** | Lingual_R. | 3.51 |
| **Lingual_L** | Lingual_R. | 3.58 |
| **Cuneus_L** | Occipital_Sup_L. | 3.18 |
| **Cuneus_R** | Occipital_Sup_L. | 3.51 |
| **Lingual_L** | Occipital_Sup_L. | 4.01 |
| **Lingual_R** | Occipital_Sup_L. | 3.36 |
| **Precentral_R** | Occipital_Sup_R. | 3.16 |
| **Supp_Motor_Area_L** | Occipital_Sup_R. | 3.27 |
| **Calcarine_R** | Occipital_Sup_R. | 3.35 |
| **Lingual_L** | Occipital_Sup_R. | 3.46 |
| **Lingual_R** | Occipital_Sup_R. | 3.26 |
| **Occipital_Sup_L** | Occipital_Sup_R. | 4.06 |
| **Rolandic_Oper_L** | Occipital_Mid_L. | 3.6 |
| **ParaHippocampal_R** | Occipital_Mid_L. | 3.47 |
| **Cuneus_L** | Occipital_Mid_L. | 3.64 |
| **Lingual_L** | Occipital_Mid_L. | 3.45 |
| **Lingual_R** | Occipital_Mid_L. | 3.34 |
| **Occipital_Sup_L** | Occipital_Mid_L. | 3.11 |
| **Occipital_Sup_R** | Occipital_Mid_L. | 3.59 |
| **Occipital_Sup_L** | Occipital_Mid_R. | 3.97 |
| **Occipital_Mid_L** | Occipital_Mid_R. | 3.11 |
| **Precentral_R** | Occipital_Inf_L. | 3.61 |
| **Frontal_Sup_L** | Occipital_Inf_L. | 3.36 |
| **Rolandic_Oper_R** | Occipital_Inf_L. | 3.16 |
| **Supp_Motor_Area_L** | Occipital_Inf_L. | 3.58 |
| **Cingulum_Ant_L** | Occipital_Inf_L. | 3.77 |
| **Cingulum_Ant_R** | Occipital_Inf_L. | 3.37 |
| **Cingulum_Mid_L** | Occipital_Inf_L. | 4.08 |
| **Cingulum_Mid_R** | Occipital_Inf_L. | 3.37 |
| **Hippocampus_L** | Occipital_Inf_L. | 4.52 |
| **Hippocampus_R** | Occipital_Inf_L. | 3.15 |
| **Amygdala_L** | Occipital_Inf_L. | 5.55 |
| **Calcarine_R** | Occipital_Inf_L. | 3.38 |
| **Cuneus_L** | Occipital_Inf_L. | 4.17 |
| **Cuneus_R** | Occipital_Inf_L. | 3.89 |
| **Occipital_Sup_L** | Occipital_Inf_L. | 4.17 |
| **Occipital_Sup_R** | Occipital_Inf_L. | 4.07 |
| **Occipital_Mid_L** | Occipital_Inf_L. | 3.1 |
| **Occipital_Mid_R** | Occipital_Inf_L. | 3.15 |
| **Supp_Motor_Area_L** | Occipital_Inf_R. | 3.68 |
| **Cingulum_Post_L** | Occipital_Inf_R. | 3.18 |
| **Hippocampus_L** | Occipital_Inf_R. | 4.07 |
| **Hippocampus_R** | Occipital_Inf_R. | 3.13 |
| **Amygdala_L** | Occipital_Inf_R. | 4.8 |
| **Calcarine_R** | Occipital_Inf_R. | 3.12 |
| **Cuneus_L** | Occipital_Inf_R. | 4.75 |
| **Occipital_Sup_L** | Occipital_Inf_R. | 3.71 |
| **Cingulum_Ant_R** | Fusiform_L. | 3.2 |
| **Cingulum_Mid_L** | Fusiform_L. | 4.37 |
| **Cingulum_Mid_R** | Fusiform_L. | 3.72 |
| **Cingulum_Post_L** | Fusiform_L. | 3.61 |
| **Cingulum_Post_R** | Fusiform_L. | 3.3 |
| **Hippocampus_L** | Fusiform_L. | 3.63 |
| **Hippocampus_R** | Fusiform_L. | 3.22 |
| **Amygdala_L** | Fusiform_L. | 5.16 |
| **Amygdala_R** | Fusiform_L. | 3.33 |
| **Calcarine_L** | Fusiform_L. | 3.62 |
| **Calcarine_R** | Fusiform_L. | 3.96 |
| **Cuneus_L** | Fusiform_L. | 3.72 |
| **Cuneus_R** | Fusiform_L. | 3.35 |
| **Lingual_L** | Fusiform_L. | 3.75 |
| **Lingual_R** | Fusiform_L. | 3.56 |
| **Occipital_Sup_L** | Fusiform_L. | 4.12 |
| **Occipital_Sup_R** | Fusiform_L. | 3.13 |
| **Occipital_Mid_L** | Fusiform_L. | 3.73 |
| **Cuneus_L** | Fusiform_R. | 3.27 |
| **Occipital_Sup_L** | Fusiform_R. | 3.22 |
| **Rolandic_Oper_L** | Postcentral_R. | 3.82 |
| **Supp_Motor_Area_L** | Postcentral_R. | 3.64 |
| **Insula_L** | Postcentral_R. | 3.23 |
| **Cingulum_Mid_L** | Postcentral_R. | 3.42 |
| **Amygdala_L** | Postcentral_R. | 3.25 |
| **Amygdala_L** | Parietal_Sup_L. | 3.78 |
| **Amygdala_R** | Parietal_Sup_L. | 3.23 |
| **Amygdala_L** | Parietal_Sup_R. | 3.49 |
| **Frontal_Inf_Oper_L** | Parietal_Inf_L. | 4.56 |
| **Frontal_Inf_Oper_R** | Parietal_Inf_L. | 4.21 |
| **Frontal_Inf_Tri_L** | Parietal_Inf_L. | 5.19 |
| **Frontal_Inf_Tri_R** | Parietal_Inf_L. | 3.63 |
| **Rolandic_Oper_L** | Parietal_Inf_L. | 4.01 |
| **Insula_L** | Parietal_Inf_L. | 3.42 |
| **Amygdala_L** | Parietal_Inf_L. | 3.59 |
| **Amygdala_L** | Parietal_Inf_R. | 3.67 |
| **Insula_L** | SupraMarginal_L. | 3.23 |
| **Parietal_Inf_L** | SupraMarginal_L. | 3.6 |
| **Rolandic_Oper_L** | SupraMarginal_R. | 5.87 |
| **Insula_L** | SupraMarginal_R. | 4.75 |
| **Parietal_Inf_L** | SupraMarginal_R. | 3.41 |
| **Frontal_Sup_R** | Angular_R. | 3.13 |
| **Cingulum_Ant_R** | Precuneus_L. | 3.46 |
| **Calcarine_R** | Paracentral_Lobule_L. | 3.25 |
| **Cingulum_Ant_R** | Paracentral_Lobule_R. | 3.19 |
| **Amygdala_L** | Paracentral_Lobule_R. | 3.9 |
| **Supp_Motor_Area_L** | Caudate_L. | 3.16 |
| **Paracentral_Lobule_L** | Caudate_L. | 3.25 |
| **Frontal_Inf_Oper_R** | Putamen_L. | 3.2 |
| **Rolandic_Oper_L** | Putamen_L. | 4.3 |
| **Rolandic_Oper_R** | Putamen_L. | 4.75 |
| **Supp_Motor_Area_L** | Putamen_L. | 4.07 |
| **Supp_Motor_Area_R** | Putamen_L. | 3.93 |
| **Fusiform_L** | Putamen_L. | 3.95 |
| **Frontal_Inf_Oper_R** | Putamen_R. | 3.15 |
| **Rolandic_Oper_L** | Putamen_R. | 5.41 |
| **Rolandic_Oper_R** | Putamen_R. | 4.24 |
| **Supp_Motor_Area_L** | Putamen_R. | 3.44 |
| **Supp_Motor_Area_R** | Putamen_R. | 3.53 |
| **Insula_L** | Putamen_R. | 3.66 |
| **Fusiform_L** | Putamen_R. | 3.45 |
| **Putamen_L** | Putamen_R. | 3.45 |
| **Rolandic_Oper_L** | Pallidum_L. | 3.41 |
| **Rolandic_Oper_R** | Pallidum_L. | 3.41 |
| **Rolandic_Oper_L** | Pallidum_R. | 4.04 |
| **Rolandic_Oper_R** | Pallidum_R. | 4.09 |
| **Fusiform_L** | Thalamus_L. | 3.62 |
| **Fusiform_L** | Thalamus_R. | 3.1 |
| **Precentral_L** | Heschl_L. | 3.29 |
| **Precentral_R** | Heschl_L. | 3.58 |
| **Frontal_Sup_R** | Heschl_L. | 3.26 |
| **Frontal_Mid_L** | Heschl_L. | 3.23 |
| **Frontal_Mid_R** | Heschl_L. | 3.61 |
| **Frontal_Inf_Oper_R** | Heschl_L. | 3.43 |
| **Supp_Motor_Area_L** | Heschl_L. | 3.63 |
| **Supp_Motor_Area_R** | Heschl_L. | 3.51 |
| **Cingulum_Ant_L** | Heschl_L. | 4.05 |
| **Cingulum_Ant_R** | Heschl_L. | 3.98 |
| **Cingulum_Mid_L** | Heschl_L. | 3.47 |
| **Cingulum_Mid_R** | Heschl_L. | 3.2 |
| **Occipital_Inf_L** | Heschl_L. | 3.86 |
| **Occipital_Inf_R** | Heschl_L. | 3.58 |
| **Postcentral_L** | Heschl_L. | 3.38 |
| **Postcentral_R** | Heschl_L. | 4.23 |
| **Parietal_Inf_L** | Heschl_L. | 3.59 |
| **SupraMarginal_R** | Heschl_L. | 4.11 |
| **Paracentral_Lobule_L** | Heschl_L. | 3.11 |
| **Putamen_L** | Heschl_L. | 3.27 |
| **Putamen_R** | Heschl_L. | 3.43 |
| **Rolandic_Oper_L** | Temporal_Sup_L. | 4.23 |
| **Rolandic_Oper_R** | Temporal_Sup_L. | 4.21 |
| **Supp_Motor_Area_L** | Temporal_Sup_L. | 3.44 |
| **Insula_L** | Temporal_Sup_L. | 4.29 |
| **Insula_R** | Temporal_Sup_L. | 3.73 |
| **SupraMarginal_R** | Temporal_Sup_L. | 3.79 |
| **Heschl_L** | Temporal_Sup_L. | 3.16 |
| **Rolandic_Oper_L** | Temporal_Sup_R. | 3.45 |
| **Rolandic_Oper_R** | Temporal_Sup_R. | 4.35 |
| **Insula_L** | Temporal_Sup_R. | 3.63 |
| **Insula_R** | Temporal_Sup_R. | 4.04 |
| **Heschl_L** | Temporal_Sup_R. | 3.17 |
| **Frontal_Sup_L** | Temporal_Pole_Sup_L. | 3.45 |
| **Rolandic_Oper_R** | Temporal_Pole_Sup_L. | 4.37 |
| **Supp_Motor_Area_L** | Temporal_Pole_Sup_L. | 3.64 |
| **Supp_Motor_Area_R** | Temporal_Pole_Sup_L. | 3.27 |
| **Cingulum_Mid_L** | Temporal_Pole_Sup_L. | 3.22 |
| **Cuneus_L** | Temporal_Pole_Sup_L. | 3.37 |
| **Rolandic_Oper_R** | Temporal_Pole_Sup_R. | 3.31 |
| **Cingulum_Mid_R** | Temporal_Pole_Sup_R. | 3.12 |
| **Cuneus_L** | Temporal_Pole_Sup_R. | 3.5 |
| **Occipital_Sup_L** | Temporal_Pole_Sup_R. | 3.47 |
| **Temporal_Pole_Sup_L** | Temporal_Pole_Sup_R. | 3.2 |
| **Frontal_Sup_Medial_L** | Temporal_Mid_L. | 3.47 |
| **Cingulum_Ant_L** | Temporal_Mid_L. | 3.13 |
| **Cingulum_Mid_L** | Temporal_Mid_L. | 3.43 |
| **Cingulum_Post_L** | Temporal_Mid_L. | 3.68 |
| **Hippocampus_L** | Temporal_Mid_L. | 3.84 |
| **Hippocampus_R** | Temporal_Mid_L. | 3.22 |
| **Amygdala_L** | Temporal_Mid_L. | 4.21 |
| **Amygdala_R** | Temporal_Mid_L. | 3.16 |
| **Cuneus_L** | Temporal_Mid_L. | 3.11 |
| **Angular_L** | Temporal_Mid_L. | 4.1 |
| **Heschl_L** | Temporal_Mid_L. | 3.4 |
| **Frontal_Inf_Tri_L** | Temporal_Mid_R. | 3.2 |
| **Frontal_Inf_Tri_R** | Temporal_Mid_R. | 3.2 |
| **Rolandic_Oper_L** | Temporal_Mid_R. | 3.23 |
| **Cingulum_Ant_R** | Temporal_Mid_R. | 3.26 |
| **Cingulum_Mid_L** | Temporal_Mid_R. | 3.22 |
| **Cingulum_Mid_R** | Temporal_Mid_R. | 3.82 |
| **Hippocampus_L** | Temporal_Mid_R. | 4.08 |
| **Hippocampus_R** | Temporal_Mid_R. | 3.76 |
| **Amygdala_L** | Temporal_Mid_R. | 5.25 |
| **Amygdala_R** | Temporal_Mid_R. | 4.32 |
| **Cuneus_L** | Temporal_Mid_R. | 4.24 |
| **Occipital_Sup_L** | Temporal_Mid_R. | 3.52 |
| **Occipital_Mid_L** | Temporal_Mid_R. | 3.6 |
| **Angular_L** | Temporal_Mid_R. | 3.44 |
| **Angular_R** | Temporal_Mid_R. | 3.62 |
| **Heschl_L** | Temporal_Mid_R. | 3.26 |
| **Frontal_Sup_L** | Temporal_Pole_Mid_L. | 3.29 |
| **Frontal_Sup_R** | Temporal_Pole_Mid_L. | 3.59 |
| **Frontal_Sup_Medial_L** | Temporal_Pole_Mid_L. | 3.4 |
| **Cingulum_Ant_R** | Temporal_Pole_Mid_L. | 3.55 |
| **Cingulum_Mid_L** | Temporal_Pole_Mid_L. | 3.58 |
| **Amygdala_L** | Temporal_Pole_Mid_L. | 3.43 |
| **Cuneus_L** | Temporal_Pole_Mid_L. | 3.4 |
| **Lingual_R** | Temporal_Pole_Mid_L. | 3.55 |
| **Occipital_Mid_L** | Temporal_Pole_Mid_L. | 3.39 |
| **Angular_L** | Temporal_Pole_Mid_L. | 4.59 |
| **Caudate_L** | Temporal_Pole_Mid_L. | 3.51 |
| **Putamen_L** | Temporal_Pole_Mid_L. | 4.85 |
| **Putamen_R** | Temporal_Pole_Mid_L. | 3.61 |
| **Thalamus_L** | Temporal_Pole_Mid_L. | 3.49 |
| **Calcarine_L** | Temporal_Pole_Mid_R. | 3.78 |
| **Calcarine_R** | Temporal_Pole_Mid_R. | 3.87 |
| **Lingual_L** | Temporal_Pole_Mid_R. | 3.51 |
| **Lingual_R** | Temporal_Pole_Mid_R. | 3.59 |
| **Occipital_Mid_L** | Temporal_Pole_Mid_R. | 3.53 |
| **Caudate_L** | Temporal_Pole_Mid_R. | 3.39 |
| **Putamen_L** | Temporal_Pole_Mid_R. | 3.23 |
| **Thalamus_L** | Temporal_Pole_Mid_R. | 3.91 |
| **Thalamus_R** | Temporal_Pole_Mid_R. | 3.35 |
| **Temporal_Pole_Mid_L** | Temporal_Pole_Mid_R. | 3.15 |
| **Frontal_Sup_L** | Temporal_Inf_L. | 3.41 |
| **Frontal_Sup_R** | Temporal_Inf_L. | 3.11 |
| **Olfactory_L** | Temporal_Inf_L. | 3.4 |
| **Frontal_Sup_Medial_L** | Temporal_Inf_L. | 3.37 |
| **Frontal_Med_Orb_R** | Temporal_Inf_L. | 3.13 |
| **Cingulum_Ant_L** | Temporal_Inf_L. | 3.45 |
| **Cingulum_Ant_R** | Temporal_Inf_L. | 3.28 |
| **Cingulum_Mid_L** | Temporal_Inf_L. | 4.52 |
| **Cingulum_Mid_R** | Temporal_Inf_L. | 3.5 |
| **Cingulum_Post_L** | Temporal_Inf_L. | 5.03 |
| **Cingulum_Post_R** | Temporal_Inf_L. | 3.55 |
| **Hippocampus_L** | Temporal_Inf_L. | 4.89 |
| **Hippocampus_R** | Temporal_Inf_L. | 3.25 |
| **Amygdala_L** | Temporal_Inf_L. | 5.51 |
| **Amygdala_R** | Temporal_Inf_L. | 3.61 |
| **Angular_L** | Temporal_Inf_L. | 3.42 |
| **Caudate_L** | Temporal_Inf_L. | 4.16 |
| **Putamen_L** | Temporal_Inf_L. | 4.38 |
| **Thalamus_L** | Temporal_Inf_L. | 3.67 |
| **Thalamus_R** | Temporal_Inf_L. | 3.18 |
| **Temporal_Pole_Sup_L** | Temporal_Inf_L. | 3.51 |
| **Cingulum_Ant_R** | Temporal_Inf_R. | 3.31 |
| **Cingulum_Mid_L** | Temporal_Inf_R. | 3.22 |
| **Cingulum_Mid_R** | Temporal_Inf_R. | 3.4 |
| **Cingulum_Post_L** | Temporal_Inf_R. | 3.27 |
| **Hippocampus_L** | Temporal_Inf_R. | 3.34 |
| **Amygdala_L** | Temporal_Inf_R. | 4.61 |
| **Calcarine_L** | Temporal_Inf_R. | 3.14 |
| **Calcarine_R** | Temporal_Inf_R. | 3.8 |
| **Cuneus_L** | Temporal_Inf_R. | 3.24 |
| **Occipital_Mid_L** | Temporal_Inf_R. | 3.22 |
| **Putamen_L** | Temporal_Inf_R. | 3.23 |
