# Supplementary material for: Functional Connectivity Changes in Behavioral, Semantic, and Nonfluent Variants of Frontotemporal Dementia
Source: Behav Neurol. 2018 Apr 1;2018:9684129. doi: 10.1155/2018/9684129 (PMC5902123; doi:10.1155/2018/9684129)
Supplement: Supplementary 4 — Table S4: networks with changes in nfvPPA + svPPA in comparison with controls. [file 9684129.f4.docx]

**Supplementary Table S4.** Results with NBS in the contrast (nfvPPA + svPPA) - Controls. The rows indicate nodes pairs with a significantly difference between previous groups.

| Node 1 | Node 2 | T-test |
| --- | --- | --- |
| Precentral_R | Rolandic_Oper_L. | 3.7 |
| Frontal_Mid_L | Rolandic_Oper_L. | 3.56 |
| Frontal_Mid_R | Rolandic_Oper_L. | 4.23 |
| Frontal_Inf_Oper_R | Rolandic_Oper_L. | 3.16 |
| Rolandic_Oper_L | Rolandic_Oper_R. | 3.26 |
| Frontal_Sup_L | Olfactory_L. | 3.12 |
| Olfactory_L | Frontal_Sup_Medial_L. | 3.18 |
| Olfactory_L | Frontal_Sup_Medial_R. | 3.46 |
| Olfactory_R | Rectus_L. | 3.23 |
| Frontal_Med_Orb_R | Rectus_L. | 3.41 |
| Precentral_R | Insula_L. | 3.48 |
| Frontal_Mid_R | Insula_L. | 3.17 |
| Rolandic_Oper_R | Insula_L. | 4.26 |
| Supp_Motor_Area_L | Insula_L. | 3.48 |
| Supp_Motor_Area_R | Insula_L. | 3.73 |
| Insula_L | Insula_R. | 4.23 |
| Rolandic_Oper_L | Cingulum_Ant_L. | 3.98 |
| Olfactory_L | Cingulum_Ant_R. | 3.24 |
| Rolandic_Oper_L | Cingulum_Mid_L. | 4.14 |
| Rolandic_Oper_R | Cingulum_Mid_L. | 3.42 |
| Rolandic_Oper_L | Cingulum_Mid_R. | 4.72 |
| Frontal_Sup_Medial_L | Cingulum_Post_L. | 3.21 |
| Frontal_Sup_Medial_R | Cingulum_Post_L. | 3.14 |
| Hippocampus_L | ParaHippocampal_L. | 3.82 |
| Hippocampus_R | ParaHippocampal_L. | 4.45 |
| Hippocampus_L | ParaHippocampal_R. | 3.52 |
| Hippocampus_R | ParaHippocampal_R. | 3.52 |
| Precentral_R | Amygdala_L. | 3.54 |
| Frontal_Sup_L | Amygdala_L. | 4.11 |
| Frontal_Sup_R | Amygdala_L. | 3.79 |
| Frontal_Mid_L | Amygdala_L. | 3.54 |
| Frontal_Mid_R | Amygdala_L. | 4.26 |
| Frontal_Inf_Orb_R | Amygdala_L. | 3.46 |
| Frontal_Sup_Medial_L | Amygdala_L. | 3.67 |
| Frontal_Sup_Medial_R | Amygdala_L. | 3.91 |
| Frontal_Mid_L | Amygdala_R. | 3.19 |
| ParaHippocampal_R | Amygdala_R. | 3.11 |
| Cingulum_Mid_L | Cuneus_L. | 3.17 |
| Calcarine_R | Cuneus_L. | 3.42 |
| Rolandic_Oper_L | Lingual_L. | 3.29 |
| Rolandic_Oper_R | Lingual_L. | 3.7 |
| Cingulum_Ant_R | Lingual_L. | 3.15 |
| Amygdala_L | Lingual_L. | 3.17 |
| Calcarine_L | Lingual_L. | 3.68 |
| Calcarine_R | Lingual_L. | 3.72 |
| Cuneus_L | Lingual_L. | 3.37 |
| Cingulum_Ant_R | Lingual_R. | 3.35 |
| Calcarine_L | Lingual_R. | 3.25 |
| Cuneus_L | Lingual_R. | 3.51 |
| Lingual_L | Lingual_R. | 3.58 |
| Cuneus_L | Occipital_Sup_L. | 3.18 |
| Cuneus_R | Occipital_Sup_L. | 3.51 |
| Lingual_L | Occipital_Sup_L. | 4.01 |
| Lingual_R | Occipital_Sup_L. | 3.36 |
| Precentral_R | Occipital_Sup_R. | 3.16 |
| Supp_Motor_Area_L | Occipital_Sup_R. | 3.27 |
| Calcarine_R | Occipital_Sup_R. | 3.35 |
| Lingual_L | Occipital_Sup_R. | 3.46 |
| Lingual_R | Occipital_Sup_R. | 3.26 |
| Occipital_Sup_L | Occipital_Sup_R. | 4.06 |
| Rolandic_Oper_L | Occipital_Mid_L. | 3.6 |
| ParaHippocampal_R | Occipital_Mid_L. | 3.47 |
| Cuneus_L | Occipital_Mid_L. | 3.64 |
| Lingual_L | Occipital_Mid_L. | 3.45 |
| Lingual_R | Occipital_Mid_L. | 3.34 |
| Occipital_Sup_L | Occipital_Mid_L. | 3.11 |
| Occipital_Sup_R | Occipital_Mid_L. | 3.59 |
| Occipital_Sup_L | Occipital_Mid_R. | 3.97 |
| Occipital_Mid_L | Occipital_Mid_R. | 3.11 |
| Precentral_R | Occipital_Inf_L. | 3.61 |
| Frontal_Sup_L | Occipital_Inf_L. | 3.36 |
| Rolandic_Oper_R | Occipital_Inf_L. | 3.16 |
| Supp_Motor_Area_L | Occipital_Inf_L. | 3.58 |
| Cingulum_Ant_L | Occipital_Inf_L. | 3.77 |
| Cingulum_Ant_R | Occipital_Inf_L. | 3.37 |
| Cingulum_Mid_L | Occipital_Inf_L. | 4.08 |
| Cingulum_Mid_R | Occipital_Inf_L. | 3.37 |
| Hippocampus_L | Occipital_Inf_L. | 4.52 |
| Hippocampus_R | Occipital_Inf_L. | 3.15 |
| Amygdala_L | Occipital_Inf_L. | 5.55 |
| Calcarine_R | Occipital_Inf_L. | 3.38 |
| Cuneus_L | Occipital_Inf_L. | 4.17 |
| Cuneus_R | Occipital_Inf_L. | 3.89 |
| Occipital_Sup_L | Occipital_Inf_L. | 4.17 |
| Occipital_Sup_R | Occipital_Inf_L. | 4.07 |
| Occipital_Mid_L | Occipital_Inf_L. | 3.1 |
| Occipital_Mid_R | Occipital_Inf_L. | 3.15 |
| Supp_Motor_Area_L | Occipital_Inf_R. | 3.68 |
| Cingulum_Post_L | Occipital_Inf_R. | 3.18 |
| Hippocampus_L | Occipital_Inf_R. | 4.07 |
| Hippocampus_R | Occipital_Inf_R. | 3.13 |
| Amygdala_L | Occipital_Inf_R. | 4.8 |
| Calcarine_R | Occipital_Inf_R. | 3.12 |
| Cuneus_L | Occipital_Inf_R. | 4.75 |
| Occipital_Sup_L | Occipital_Inf_R. | 3.71 |
| Cingulum_Ant_R | Fusiform_L. | 3.2 |
| Cingulum_Mid_L | Fusiform_L. | 4.37 |
| Cingulum_Mid_R | Fusiform_L. | 3.72 |
| Cingulum_Post_L | Fusiform_L. | 3.61 |
| Cingulum_Post_R | Fusiform_L. | 3.3 |
| Hippocampus_L | Fusiform_L. | 3.63 |
| Hippocampus_R | Fusiform_L. | 3.22 |
| Amygdala_L | Fusiform_L. | 5.16 |
| Amygdala_R | Fusiform_L. | 3.33 |
| Calcarine_L | Fusiform_L. | 3.62 |
| Calcarine_R | Fusiform_L. | 3.96 |
| Cuneus_L | Fusiform_L. | 3.72 |
| Cuneus_R | Fusiform_L. | 3.35 |
| Lingual_L | Fusiform_L. | 3.75 |
| Lingual_R | Fusiform_L. | 3.56 |
| Occipital_Sup_L | Fusiform_L. | 4.12 |
| Occipital_Sup_R | Fusiform_L. | 3.13 |
| Occipital_Mid_L | Fusiform_L. | 3.73 |
| Cuneus_L | Fusiform_R. | 3.27 |
| Occipital_Sup_L | Fusiform_R. | 3.22 |
| Rolandic_Oper_L | Postcentral_R. | 3.82 |
| Supp_Motor_Area_L | Postcentral_R. | 3.64 |
| Insula_L | Postcentral_R. | 3.23 |
| Cingulum_Mid_L | Postcentral_R. | 3.42 |
| Amygdala_L | Postcentral_R. | 3.25 |
| Amygdala_L | Parietal_Sup_L. | 3.78 |
| Amygdala_R | Parietal_Sup_L. | 3.23 |
| Amygdala_L | Parietal_Sup_R. | 3.49 |
| Frontal_Inf_Oper_L | Parietal_Inf_L. | 4.56 |
| Frontal_Inf_Oper_R | Parietal_Inf_L. | 4.21 |
| Frontal_Inf_Tri_L | Parietal_Inf_L. | 5.19 |
| Frontal_Inf_Tri_R | Parietal_Inf_L. | 3.63 |
| Rolandic_Oper_L | Parietal_Inf_L. | 4.01 |
| Insula_L | Parietal_Inf_L. | 3.42 |
| Amygdala_L | Parietal_Inf_L. | 3.59 |
| Amygdala_L | Parietal_Inf_R. | 3.67 |
| Insula_L | SupraMarginal_L. | 3.23 |
| Parietal_Inf_L | SupraMarginal_L. | 3.6 |
| Rolandic_Oper_L | SupraMarginal_R. | 5.87 |
| Insula_L | SupraMarginal_R. | 4.75 |
| Parietal_Inf_L | SupraMarginal_R. | 3.41 |
| Frontal_Sup_R | Angular_R. | 3.13 |
| Cingulum_Ant_R | Precuneus_L. | 3.46 |
| Calcarine_R | Paracentral_Lobule_L. | 3.25 |
| Cingulum_Ant_R | Paracentral_Lobule_R. | 3.19 |
| Amygdala_L | Paracentral_Lobule_R. | 3.9 |
| Supp_Motor_Area_L | Caudate_L. | 3.16 |
| Paracentral_Lobule_L | Caudate_L. | 3.25 |
| Frontal_Inf_Oper_R | Putamen_L. | 3.2 |
| Rolandic_Oper_L | Putamen_L. | 4.3 |
| Rolandic_Oper_R | Putamen_L. | 4.75 |
| Supp_Motor_Area_L | Putamen_L. | 4.07 |
| Supp_Motor_Area_R | Putamen_L. | 3.93 |
| Fusiform_L | Putamen_L. | 3.95 |
| Frontal_Inf_Oper_R | Putamen_R. | 3.15 |
| Rolandic_Oper_L | Putamen_R. | 5.41 |
| Rolandic_Oper_R | Putamen_R. | 4.24 |
| Supp_Motor_Area_L | Putamen_R. | 3.44 |
| Supp_Motor_Area_R | Putamen_R. | 3.53 |
| Insula_L | Putamen_R. | 3.66 |
| Fusiform_L | Putamen_R. | 3.45 |
| Putamen_L | Putamen_R. | 3.45 |
| Rolandic_Oper_L | Pallidum_L. | 3.41 |
| Rolandic_Oper_R | Pallidum_L. | 3.41 |
| Rolandic_Oper_L | Pallidum_R. | 4.04 |
| Rolandic_Oper_R | Pallidum_R. | 4.09 |
| Fusiform_L | Thalamus_L. | 3.62 |
| Fusiform_L | Thalamus_R. | 3.1 |
| Precentral_L | Heschl_L. | 3.29 |
| Precentral_R | Heschl_L. | 3.58 |
| Frontal_Sup_R | Heschl_L. | 3.26 |
| Frontal_Mid_L | Heschl_L. | 3.23 |
| Frontal_Mid_R | Heschl_L. | 3.61 |
| Frontal_Inf_Oper_R | Heschl_L. | 3.43 |
| Supp_Motor_Area_L | Heschl_L. | 3.63 |
| Supp_Motor_Area_R | Heschl_L. | 3.51 |
| Cingulum_Ant_L | Heschl_L. | 4.05 |
| Cingulum_Ant_R | Heschl_L. | 3.98 |
| Cingulum_Mid_L | Heschl_L. | 3.47 |
| Cingulum_Mid_R | Heschl_L. | 3.2 |
| Occipital_Inf_L | Heschl_L. | 3.86 |
| Occipital_Inf_R | Heschl_L. | 3.58 |
| Postcentral_L | Heschl_L. | 3.38 |
| Postcentral_R | Heschl_L. | 4.23 |
| Parietal_Inf_L | Heschl_L. | 3.59 |
| SupraMarginal_R | Heschl_L. | 4.11 |
| Paracentral_Lobule_L | Heschl_L. | 3.11 |
| Putamen_L | Heschl_L. | 3.27 |
| Putamen_R | Heschl_L. | 3.43 |
| Rolandic_Oper_L | Temporal_Sup_L. | 4.23 |
| Rolandic_Oper_R | Temporal_Sup_L. | 4.21 |
| Supp_Motor_Area_L | Temporal_Sup_L. | 3.44 |
| Insula_L | Temporal_Sup_L. | 4.29 |
| Insula_R | Temporal_Sup_L. | 3.73 |
| SupraMarginal_R | Temporal_Sup_L. | 3.79 |
| Heschl_L | Temporal_Sup_L. | 3.16 |
| Rolandic_Oper_L | Temporal_Sup_R. | 3.45 |
| Rolandic_Oper_R | Temporal_Sup_R. | 4.35 |
| Insula_L | Temporal_Sup_R. | 3.63 |
| Insula_R | Temporal_Sup_R. | 4.04 |
| Heschl_L | Temporal_Sup_R. | 3.17 |
| Frontal_Sup_L | Temporal_Pole_Sup_L. | 3.45 |
| Rolandic_Oper_R | Temporal_Pole_Sup_L. | 4.37 |
| Supp_Motor_Area_L | Temporal_Pole_Sup_L. | 3.64 |
| Supp_Motor_Area_R | Temporal_Pole_Sup_L. | 3.27 |
| Cingulum_Mid_L | Temporal_Pole_Sup_L. | 3.22 |
| Cuneus_L | Temporal_Pole_Sup_L. | 3.37 |
| Rolandic_Oper_R | Temporal_Pole_Sup_R. | 3.31 |
| Cingulum_Mid_R | Temporal_Pole_Sup_R. | 3.12 |
| Cuneus_L | Temporal_Pole_Sup_R. | 3.5 |
| Occipital_Sup_L | Temporal_Pole_Sup_R. | 3.47 |
| Temporal_Pole_Sup_L | Temporal_Pole_Sup_R. | 3.2 |
| Frontal_Sup_Medial_L | Temporal_Mid_L. | 3.47 |
| Cingulum_Ant_L | Temporal_Mid_L. | 3.13 |
| Cingulum_Mid_L | Temporal_Mid_L. | 3.43 |
| Cingulum_Post_L | Temporal_Mid_L. | 3.68 |
| Hippocampus_L | Temporal_Mid_L. | 3.84 |
| Hippocampus_R | Temporal_Mid_L. | 3.22 |
| Amygdala_L | Temporal_Mid_L. | 4.21 |
| Amygdala_R | Temporal_Mid_L. | 3.16 |
| Cuneus_L | Temporal_Mid_L. | 3.11 |
| Angular_L | Temporal_Mid_L. | 4.1 |
| Heschl_L | Temporal_Mid_L. | 3.4 |
| Frontal_Inf_Tri_L | Temporal_Mid_R. | 3.2 |
| Frontal_Inf_Tri_R | Temporal_Mid_R. | 3.2 |
| Rolandic_Oper_L | Temporal_Mid_R. | 3.23 |
| Cingulum_Ant_R | Temporal_Mid_R. | 3.26 |
| Cingulum_Mid_L | Temporal_Mid_R. | 3.22 |
| Cingulum_Mid_R | Temporal_Mid_R. | 3.82 |
| Hippocampus_L | Temporal_Mid_R. | 4.08 |
| Hippocampus_R | Temporal_Mid_R. | 3.76 |
| Amygdala_L | Temporal_Mid_R. | 5.25 |
| Amygdala_R | Temporal_Mid_R. | 4.32 |
| Cuneus_L | Temporal_Mid_R. | 4.24 |
| Occipital_Sup_L | Temporal_Mid_R. | 3.52 |
| Occipital_Mid_L | Temporal_Mid_R. | 3.6 |
| Angular_L | Temporal_Mid_R. | 3.44 |
| Angular_R | Temporal_Mid_R. | 3.62 |
| Heschl_L | Temporal_Mid_R. | 3.26 |
| Frontal_Sup_L | Temporal_Pole_Mid_L. | 3.29 |
| Frontal_Sup_R | Temporal_Pole_Mid_L. | 3.59 |
| Frontal_Sup_Medial_L | Temporal_Pole_Mid_L. | 3.4 |
| Cingulum_Ant_R | Temporal_Pole_Mid_L. | 3.55 |
| Cingulum_Mid_L | Temporal_Pole_Mid_L. | 3.58 |
| Amygdala_L | Temporal_Pole_Mid_L. | 3.43 |
| Cuneus_L | Temporal_Pole_Mid_L. | 3.4 |
| Lingual_R | Temporal_Pole_Mid_L. | 3.55 |
| Occipital_Mid_L | Temporal_Pole_Mid_L. | 3.39 |
| Angular_L | Temporal_Pole_Mid_L. | 4.59 |
| Caudate_L | Temporal_Pole_Mid_L. | 3.51 |
| Putamen_L | Temporal_Pole_Mid_L. | 4.85 |
| Putamen_R | Temporal_Pole_Mid_L. | 3.61 |
| Thalamus_L | Temporal_Pole_Mid_L. | 3.49 |
| Calcarine_L | Temporal_Pole_Mid_R. | 3.78 |
| Calcarine_R | Temporal_Pole_Mid_R. | 3.87 |
| Lingual_L | Temporal_Pole_Mid_R. | 3.51 |
| Lingual_R | Temporal_Pole_Mid_R. | 3.59 |
| Occipital_Mid_L | Temporal_Pole_Mid_R. | 3.53 |
| Caudate_L | Temporal_Pole_Mid_R. | 3.39 |
| Putamen_L | Temporal_Pole_Mid_R. | 3.23 |
| Thalamus_L | Temporal_Pole_Mid_R. | 3.91 |
| Thalamus_R | Temporal_Pole_Mid_R. | 3.35 |
| Temporal_Pole_Mid_L | Temporal_Pole_Mid_R. | 3.15 |
| Frontal_Sup_L | Temporal_Inf_L. | 3.41 |
| Frontal_Sup_R | Temporal_Inf_L. | 3.11 |
| Olfactory_L | Temporal_Inf_L. | 3.4 |
| Frontal_Sup_Medial_L | Temporal_Inf_L. | 3.37 |
| Frontal_Med_Orb_R | Temporal_Inf_L. | 3.13 |
| Cingulum_Ant_L | Temporal_Inf_L. | 3.45 |
| Cingulum_Ant_R | Temporal_Inf_L. | 3.28 |
| Cingulum_Mid_L | Temporal_Inf_L. | 4.52 |
| Cingulum_Mid_R | Temporal_Inf_L. | 3.5 |
| Cingulum_Post_L | Temporal_Inf_L. | 5.03 |
| Cingulum_Post_R | Temporal_Inf_L. | 3.55 |
| Hippocampus_L | Temporal_Inf_L. | 4.89 |
| Hippocampus_R | Temporal_Inf_L. | 3.25 |
| Amygdala_L | Temporal_Inf_L. | 5.51 |
| Amygdala_R | Temporal_Inf_L. | 3.61 |
| Angular_L | Temporal_Inf_L. | 3.42 |
| Caudate_L | Temporal_Inf_L. | 4.16 |
| Putamen_L | Temporal_Inf_L. | 4.38 |
| Thalamus_L | Temporal_Inf_L. | 3.67 |
| Thalamus_R | Temporal_Inf_L. | 3.18 |
| Temporal_Pole_Sup_L | Temporal_Inf_L. | 3.51 |
| Cingulum_Ant_R | Temporal_Inf_R. | 3.31 |
| Cingulum_Mid_L | Temporal_Inf_R. | 3.22 |
| Cingulum_Mid_R | Temporal_Inf_R. | 3.4 |
| Cingulum_Post_L | Temporal_Inf_R. | 3.27 |
| Hippocampus_L | Temporal_Inf_R. | 3.34 |
| Amygdala_L | Temporal_Inf_R. | 4.61 |
| Calcarine_L | Temporal_Inf_R. | 3.14 |
| Calcarine_R | Temporal_Inf_R. | 3.8 |
| Cuneus_L | Temporal_Inf_R. | 3.24 |
| Occipital_Mid_L | Temporal_Inf_R. | 3.22 |
| Putamen_L | Temporal_Inf_R. | 3.23 |
